# Continuous and Pulsed Ultraviolet-C LED on Germicidal Effect and Energy Consumption in Fresh Products: Applications in Tomatoes as a Model

**DOI:** 10.3390/foods11223636

**Published:** 2022-11-14

**Authors:** Eduardo Flores Gallegos, Nivia Escalante-García, Daniel Alanis-Lumbreras, Rumen Ivanov-Tsonchev, Alfredo Lara-Herrera, Ernesto Olvera-Gonzalez

**Affiliations:** 1 Doctorado en Ciencias de la Ingeniería, Unidad Académica de Ingeniería Eléctrica, Campus UAZ Siglo XX1, Universidad Autónoma de Zacatecas, Zacatecas 98160, Mexico; 2 Laboratorio de Iluminación Artificial, Tecnológico Nacional de México Campus Pabellón de Arteaga, Aguascalientes 20670, Mexico; 3 Unidad Académica de Física, Universidad Autónoma de Zacatecas, Zacatecas 98060, Mexico; 4 Unidad Académica de Agronomía, Universidad Autónoma de Zacatecas, Zacatecas 98170, Mexico

**Keywords:** continuous and pulsed UV-C LED light, mathematical model, energy savings, disinfection process, food safety

## Abstract

Nowadays, lifestyle change is one of the problems of the new world economic order, and the procedures of feeding, purchasing, preparation, and the storage of food products, are forcing authorities to establish more rigorous methods concerning the control of food quality and safety. Owing quality in the agro-food sector is a complex and global issue, due to the distance between production and final consumption, as well as the new demands of society on food. Contributing to the bacteria minimization during their path in the supply chain, the objective of this research is the use of an UV-C LED artificial lighting system with emission in continuous light (CL) and two of pulsed light (Mode 1 and Mode 2) for fresh products’ disinfection. A mathematical model is introduced as a reference to establish the equivalence dose of continuous and pulsed UV-C LED irradiation. The doses applied were 5, 15, and 25 mJ cm^−^². The configured parameters per each technique were the irradiance, time also the frequency (500 Hz), and duty cycle (30, 50, and 80%) for Mode 1 and Mode 2. The germicidal effect (GE), energy consumption, and effective germicidal effect (EGE), were evaluated for the different techniques. According to the results, the technique Mode 1 was the best in the GE with 1.06 ± 0.01 and 1.08 ± 0.01 Log reduction by 25 mJ cm^−2^ at 30 and 80% duty cycle, correspondingly. The CL and Mode 1 showed an outstanding performance with the EGE. Finally, Mode 1 reduced 11% in energy and the GE is comparable with CL. The pulsed light technique Mode 1 constitutes a powerful method against the microorganism’s destruction and a strategy for saving energy during the treatment. The UV-C LEDs proved to be an excellent alternative in the disinfection of fresh products with pulsed light emission in the real process.

## 1. Introduction

New trends in food consumption at the global, national, and regional levels, are focused on product demand that meets safety and quality standards. When referring to food contamination, we address chemical, physical, or biological contamination. The last is caused by different pathogens or microorganisms that include food poisoning, which originates from a large number of diseases, and is considered one of the main public health problems. Food contamination can be introduced during any point, procedure, operation, or stage of the food chain containing raw materials, from primary production to final consumption, directly affecting consumers [1].

At the same time, the current demand for fresh and easy-to-prepare products, particularly fruits and vegetables, has brought about the establishment of new disinfection methods [2]. Ultraviolet (UV) Light application in the food irradiation process is an alternative method to the conventional disinfection process and has been used for water disinfection since the beginning of the 20th century [3].

Traditional UV lamps (xenon and mercury) have several disadvantages that have not allowed for exploiting UV technology as a food decontamination method. Mercury lamps have been used as the main source for continuous wave (CW) treatments. Depending on the mercury vapor pressure during functioning, these lamps can be either low-pressure LP (254 nm) or medium-pressure MP (200–600 nm) [4]. 

Although their germicidal effect has been demonstrated, they also present disadvantages such as: short lifetime; the possibility of mercury contamination if they break; heating; accumulated dirt during disinfection; and the inability to pulse at high frequencies [5,6]. Another mercury lamps drawback is the possibility of overheating the food during the irradiation process [7]. Additionally, xenon lamps can be configured to apply pulsed light (PL) to reach doses equivalent to those of continuous wave (mercury lamps). PL is defined as short time pulses of an intense broad spectrum; the high intensity of UV-C light by increasing the energy for short periods [8]. Their efficiency has been tested in many PL studies [9]. However, the PL emitted by xenon lamps also present different disadvantages, such as: high energy demand; generating a broad spectrum (200–1100 nm); higher maintenance; slow response speed; and in some cases, it produces overheating in food [10]. 

Different mercury and xenon lamp experiments have been presented in the literature to determine the relationship in the doses and the maximum emitted power on the germicidal effect. Levy et al. [11] evaluated the UV-C pulsed light germicidal effect on continuous light by setting different doses, and microorganisms types, on environmental elements. The doses used were various in a range from 0.17 J cm^−2^ to 5.82 J cm^−2^, from one to 10 pulses during a period of 1 s, and were emitted with variable voltage inputs from 3 kV to 1 kV. Cheigh et al. [12] equipped a laboratory scale PL using xenon lamps to produce intense pulsed light (IPL). They analyzed the germicidal effect of IPL versus UV-C irradiation on pathogenic *L. monocytogenes* on seafood products, as well as culture media contaminated with this type of bacteria. Different doses (0.11–1.75 mJ cm^−2^ for 1.5 µs at 5 Hz frequency) in a pulse range for periods from 0–1960 s, as well as 0 to 17.2 mJ cm^−2^ total fluence, were applied at a distance between the sample and the system of 80 mm. Bohrerova et al. [13] experimented with the efficiency of *E. coli* microorganisms disinfection using pulsed UV irradiation, compared to continuous wave UV irradiation tools. 

Other research obtained a similar germicidal effect through photochemical processes. This technique presents higher power in shorter exposure time, or lower intensity with longer exposure time [14]. An UV laser lamp emission at doses 35 and 250 J m^−2^ per pulse compared through low power continuous mercury lamp of 90 W m^−2^ output on the inactivation of *Bacillus subtiliss* spores. The amount of photons per surface unit was considered as a variable in order to find differences between the emission of pulsed light [15]. Inhibition on *E. coli* was evaluated by applying high energy ultraviolet radiation with 30 ms pulses using a xenon flash lamp. A pyroelectric detector was used to establish the doses, thus measuring the amount of energy at each wavelength [16]. Another proposal evaluated the different microorganism’s species inactivation with high intensity ultraviolet light with pulses of 325 µs duration in a range of 190 to 1.00 nm (including the regions of the UV−A, B and C−spectrum, in different percentages). The total influence was calculated according to the number of light pulses emitted (0.14–12 J cm^−2^) [17]. 

Recently, the application of Ultraviolet Light-Emitting Diodes (UV-LEDs) is currently being increased, because of their many advantages, including: as the germicidal power in food high; efficiency in energy-saving; environmentally friendly; contains no gases metals, or toxic substances; constant light intensity; management in heat up and temperature; a longer lifetime; and including the application of light in specific bands (λ) of the UV spectrum (210 to 400 nm). A key point raised is that the emission peaks of UV-LEDs can be used as single or mixed to generate an optimal synergy over the inactivation of a specific microorganism, or for a variety of pathogens [18,19]. 

Additionally, the UV-C LEDs require less power, and offer a wider range of pulse repetition frequency, pulse lengths, and duty cycles [20]. That is, UV-LEDs can flicker or flash in short periods in which the lamp emission is turned on/off in fast intervals (µs) producing pulsed light of high intensity [21]. Moreover, to these advantages, the United Nations through the United Nations Environment Program (UNEP) established the Minamata Convention in which they propose to discontinue mercury lamps and choose a new UV emitting source [22]. Different investigations with PL with LEDs have been developed by experimenting with parameters such as frequency or duty cycle. Green et al. [23] conducted a comparative study on the effectiveness and performance of UV-LEDs (emission at 259, 268, 275, 289, and 370) with a mercury lamp (low pressure at 253.7 nm) on several foodborne pathogens with a dose equivalent to 7 mJ/cm^2^. Findings with UV-LEDs at 259 and 268 nm were superior compared to the mercury lamp. Sholtes and Linden [6] conducted a comparison with mercury lamps (emitted range 0.05–0.638 mW cm^−2^) and UV-LEDs (16 to 60 mJ cm^−2^) on *E. coli* and *P. aeruginosa*. The authors’ proposal has an interesting point to evaluate the germicidal effect in the different treatments, the energy consumption taken into account. Different microorganism’s inhibition with UV-LEDs through continuous and pulsed light emissions for water disinfection, together with different frequencies (0.1, 1, 1, 10, 100, 1 k Hz) and duty cycles (10, 25, 50, 75, and 90%), were set for pulsed irradiation. The authors adjusted the exposure time according to each configuration to ensure equivalence, making use of actinometry [24]. Nyangaresi et al. [25] compared continuous and pulsed UV-LED light. They indicated that pulsed light management reduces the heating of UV-LEDs. The dose calculated as the product of irradiance (mW cm^−2^), exposure time (s), and duty cycle (%). For continuous light emission, the duty cycle is at 100%. Zou et al. [21] evaluated the continuous and pulsed UV-LED light effectiveness for deactivation of *E. coli* in water. The authors applied several duty cycles (5, 10, 20, 50, and 100) at 1 K Hz frequency for the dose of 8.4 mJ cm^−2^. Nyhan et al. [26] experimented using UV-LEDs in four bacteria inactivation on plastic surfaces and powdered ingredients (onion powder, garlic powder, cheese powder, onion powder, and chili powder). Mercury lamps and UV-LEDs (A and C) were compared over the germicidal effect. UV-C LEDs used continuous light emission in the range of 250–280 nm to skinless chicken, stainless steel, and high-density polyethylene contaminated with *Salmonella Enterica*. The amount of irradiation applied was 2 mW/cm^2^ (50%) or 4 mW/cm^2^ (100%), varying the time. The results obtained were 1.02 and 1.78 Log [27].

**Figure 1:**
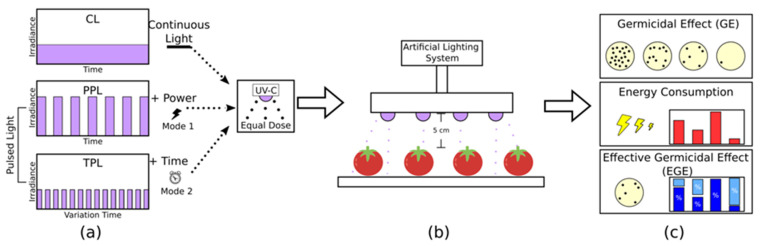
General scheme of the experiment. (**a**) UV-C LED techniques: Continuous light (CL), power pulsed light (PPL), time pulsed light (TPL). (**b**) Fresh products disinfection. (**c**) Data evaluation.

This proposal presents a germicidal effect comparison and energy consumption evaluation with continuous light (CL) and two pulsed light (Power Pulsed Light–PPL) or Mode 1- and Time Pulsed Light–TPL or Mode 2–different techniques. Figure 1 represents the techniques used for all doses (5, 15, and 25 mJ cm^−2^) with parameters such as irradiance, time, and frequency (500 Hz) and duty cycle (30, 50, and 80%) for PL as initial parameters. All the techniques achieved the same dose through the proposed mathematical model. This research constituted a powerful tool for fresh food disinfection. UV-C LEDs in pulsed light showed to be an alternative to improving the sanitary conditions of fresh foods. Additionally, UV-LEDs have a high potential for the food industry due to their unique characteristics and the possibility of being incorporated into a wide variety of disinfection systems.

## 2. Materials and Methods

Figure 1 represents the general configuration, the first step is to establish the energy equivalence between continuous and pulsed irradiation, that is, the same dose in the different techniques of light emitted on fresh produce. A second step, the parameters in the artificial lighting system for Mode 1 and Mode 2, are configured for tomato disinfection. The third step is to evaluate the germicidal effect (GE), energy consumption, and effective germicidal effect (EGE) for each light technique.

### 2.1. UV-C LEDs Artificial Lighting System Characteristics

The UV-C LEDs lighting system design and manufacture were developed by the Artificial Lighting Laboratory (LIA) at Instituto Tecnológico de Pabellón de Arteaga in Aguascalientes, México. We used UV-C LEDs (a wavelength of 268 nm) at 25 watts each. The lamps include properties for switching the emission light in continuous and pulsed mode, the frequency, and duty cycles. The automated controller based on Field Programmable Gate Array (FPGA) allows programming different functions such as pulse frequency, duty cycle, intensity emitted, wavelength, and on-off time, plus additional characteristic for the lighting system. An aluminum lamp with fans to dissipate the heat were installed in UV-C LEDs. An array of three lamps were used. 

### 2.2. UV-C LEDs Characterization

The UV-C LEDs lighting system (an array of three lamps) was characterized by a Spectroradiometer ILT950UV (International Light Technologies, Massachusetts, United States) ranging from 200 to 1100 nm. The sensor is placed parallel to the artificial lighting system to obtain information. The UV-C LEDs emitted an effective zone around 100 cm^2^ (10 × 10 cm) to set the tomatoes. A distance of 5 cm in the lamps and the product was configured. 

### 2.3. Dose Setup

The dosage definition is the base of all techniques proposed. The dose programming includes the time and irradiance values of the UV-C LED lamps. The doses are determined by Equation (1) [3]:

(1) D=I×t

where *D* refers to the doses applied expressed in J cm^−2^; *I* represent the irradiance in mW cm^−2^; *t* is the time in seconds (s).

The doses configured for this experiment were 5, 15, and 25 mJ cm^−2^.

### 2.4. Mathematical Model for the UV-C LEDs Doses Techniques for Continuous and Pulsed Light

**Figure 2:**
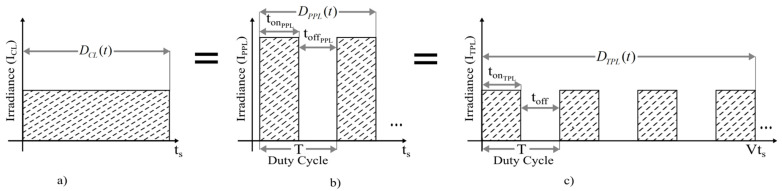
UV-C LEDs emission techniques. (**a**) Continuous light (CL). (**b**) Power pulsed light (PPL). (**c**) Time pulsed light (TPL).

Figure 2 describes the energy equivalence configuration (same irradiance) applied for the disinfection of fresh food by UV-C LEDs. We presented techniques for continuous and pulsed UV-C LED light to evaluate the germicidal effect (GE) and energy consumption, and the effective germicidal effect during the disinfection process. The dose in all modes depends on irradiance and emission time, according to Equation (1). Continuous light (CL, Figure 2a) is the emission of energy (photons) or spectral distribution over a period (time) for the fresh products disinfection. Pulsed light (Mode 1 and Mode 2) the dose set through different frequencies (number of cycles per second), duty cycles (percent of periodicity spent on), and time emitted by the artificial illumination system. PPL dose (Mode 1, Figure 2b) changes to reach the potential difference (amplitude or voltage) of the electric current through different pulses within the same time programmed for CL. TPL (Mode 2) maintains the CL irradiation parameter at frequency and duty cycle for Mode 1. Meanwhile, the time is prolonged at different duration to achieve the same energy (number of photons) emitted by the artificial lighting system in CL. We expressed the mathematical models below.

#### 2.4.1. Continuous Light (CL)

UV-C LED continuous light is graphically represented in Figure 2a; the dose in continuous light refers to the electric energy in photons (area under the curve) supply to the disinfection tomatoes during a time (t). Irradiance ($I_{\mathrm{CL}}$) defines the incident power per unit area, that is, the average amount of incident energy per unit area and unit time [28].

The continuous light is used commonly in the germicidal inactivation and depends completely on the number of seconds to fresh products are exposed. The dose ($D_{\mathrm{CL}}$) programming includes the values of the time experiment (*t*) and the irradiance ($I_{\mathrm{CL}}$) in continuous mode. The CL technique is described as Equation (2).

(2) $$D_{\mathrm{CL}}(t)=\int_{0}^{t_{\mathrm{CL}}}I_{\mathrm{CL}}\times t\;dt$$

where $D_{\mathrm{CL}}$ is the dose configured during the experiment; $I_{\mathrm{CL}}$ defines the amount of radiation emitted by the UV-C LED lamps; t represents the time during the emission irradiation on continuous light; $t_{\mathrm{CL}}$ is the period of time in seconds on continuous light.

#### 2.4.2. Mode 1 (PPL)

The pulse light in Figure 2a,b is the energy equivalence of continuous light and is obtained by calculating the sum of the area under the curve of each mode in pulsed light. In pulsed light emission, it is possible to control the intensity depending on the parameters of frequency, pulse duration (period), and duty cycle. This technique individual irradiation pulses have a specific time, intensity, and spectral distribution allowing for a controlled and confined energy delivery into the fresh product. $t_{{\mathrm{on}}_{\mathrm{PPL}}}$ exists in the same period (1/f) established for the treatment time. A point to consider is that if the irradiance level is continuously modified (the parameter CL), the signal amplitude for pulsed light (Mode 1) is adjusted to achieve energy equivalence between the two modes of operation. This concept is defined according to Equation (3):

(3) $$D_{\mathrm{PPL}}(t)=N_{p}\int_{0}^{t_{{\mathrm{on}}_{{}_{\mathrm{PPL}}}}}I_{\mathrm{PPL}}dt$$

$D_{\mathrm{PPL}}$ refers to the doses applied expressed in J∙cm^−2^. The equations required to perform the pulsed modes are given below.

(4) $$I_{\mathrm{PPL}}=\frac{I_{\mathrm{CL}}\times 100}{DutyCycle}$$

$I_{\mathrm{PPL}}$ is the amount e radiation emitted by the UV-C LED lamps in Power Pulsed Light depends on the *I_CL_* as the Equation (4).

(5) $$N_{p}=f\times t$$

$N_{p}$ represents the number of pulses where *f* is equal to the frequency in Hertz (Hz) and *t* is the time in seconds (s) when the lamps are turned on and off.

(6) $$t_{{\mathrm{on}}_{\mathrm{PPL}}},t_{{\mathrm{on}}_{\mathrm{TPL}}}=\frac{DutyCycle\times T}{100}$$

Time on ($t_{{\mathrm{on}}_{\mathrm{PPL}}},t_{{\mathrm{on}}_{\mathrm{TPL}}}$) in seconds, that is, the light turned on in the pulsed light depends on the duty cycle and the frequency. Period (T) depends on the selected pulsed frequency. The value of frequency for this case remains constant at 500 Hz.

#### 2.4.3. Mode 2 (TPL) 

During each period there is only one pulse delivering all the energy emitted for the duration of time. However, to operate the UV-C LED irradiation in Time Pulsed Light (TPL or Mode 2) while maintaining the parameters of the continuous mode (CL) and to ensure energy equivalence (same amount of irradiation). The Mode 2 is represented by the Equation (7):

(7) $$D_{\mathrm{TPL}}(t)=v_{t}\times N_{p}\int_{0}^{t_{{\mathrm{on}}_{{}_{\mathrm{TPL}}}}}I_{\mathrm{TPL}}dt$$

where $D_{\mathrm{TPL}}$ refers to the doses applied expressed in J∙cm^−2^. $I_{\mathrm{TPL}}$ is the radiation magnitude emitted by the UV-C LED lamps in Time Pulsed Light; $N_{p}$ represents the number of pulses (the pulses number is equal to the frequency (*f*) in Hertz times the treatment time; $t_{{\mathrm{on}}_{{}_{\mathrm{TPL}}}}$ represent the time in seconds and depends on the duty cycle, frequency, and both are set as initial parameters, calculated with the Equation (6); $v_{t}$ is the factor of 100 divided by the duty cycle (Equation (8)).

(8) $$v_{t}=\frac{100}{DutyCycle}$$

### 2.5. Fresh Product Material and Experimental Design

Fresh tomatoes serve to validate the germicidal effect of UV-C LEDs. The product was taken from the same harvest from a local retailer. Before the irradiation process, we determined the initial number of microorganisms (Aerobic Mesophilic, AM). The experiments were based on a completely randomized design, and results are expressed as mean ± standard deviation or ± standard error. For all conditions, four repetitions were carried out. A Shapiro-Wilks test was performed to determine the normality of the data. Subsequently, for the validation of the germicidal effect, one analysis of variance (ANOVA) was performed (*p* < 0.05) to determine if there are significant germicidal differences between doses or techniques. Statistical tests were performed in the R software.

### 2.6. Organism and Counting Approach 

The microorganisms analyzed for the experimentation were aerobic mesophilic (AM) to verify the sanitary quality of food, the handling conditions, and hygienic conditions [24]. We employed 3M™ Petri film Aerobic Count plates, for eliminating the time-consuming of preparing media/agar dishes. One hour before the counting procedure, the AM plates were hydrated with peptone (one milliliter). Colony-Forming Unit (CFU) were counted per sample (six tomatoes). The same procedure was applied in all samples. The Germicidal Effect (GE) was analyzed by the Equation (9):

(9) $$\mathrm{Log}GE=\mathrm{Log}\left(\frac{N_{0}}{N}\right)$$

where *N*_0_ and *N* are the colony count (CFU) before and after process disinfection, respectively. 

### 2.7. Effective Germicidal Effect 

The effective germicidal effect (*EGE*) performance is the difference in germicidal efficacy over the period (time) or dose. That is, the *EGE* represents the difference in germicidal effect per technique. The concept examines and optimizes the energy consumption in the artificial radiation system considering also the germicidal effect.

The *EGE* unit parameter is in proportion (%) and represented as Equation (10):

(10) $$EGE=N-N_{0}$$

where *EGE* represents the effective germicidal effect in percentage (%), *N*_0_ and *N* are the colony count (CFU) before and after process irradiation, respectively.

### 2.8. Energy Consumption 

Power consumption was determined with a wattmeter for each of the treatments. The data are shown in Watts per Technique (*W*/Technique). At the end of each treatment, the energy consumption was measured with a hook-on AC ammeter (Peak Teach, Salerno, Italy) and is expressed in *W* using the following relation in Equation (11):

(11) W=A×V×t

where *V* is equal to the potential difference in volts (V), that is, the energy consumed by the lamps during technique. *A* refers to the flow of electricity as an electric current in amperes (A). *t* is the time in seconds (s).

## 3. Results and Discussion

The disinfection process was carried out with UV-C LEDs irradiation source in continuous and pulsed light (Mode 1 and Mode 2). The established techniques were tested on fresh tomatoes. The continuous and pulsed light configured at the same conditions (temperature, irradiation area, intensity). The frequency used for the Mode 1 and Mode 2 (UV-C pulsed light) at 500 Hz with different duty cycles at 30, 50, and 80%. The data obtained are presented in this section. 

### 3.1. UV-C LEDs Characterization for Continuous and Pulsed Light Techniques

Table 1 displays the obtained values for the irradiation and time according to the configured parameters such as dose, frequency and duty cycle through the spectroradiometer. This step is crucial to be allowed to specify an equivalent dose between the different techniques. The continuous light is applied as a reference parameter for the total energy consumption per light technique. When configuring pulsed light (Mode 1 and Mode 2) it is necessary to obtain the energy equivalence through the mathematical model, that is, to calculate the intensity or irradiance and the time of pulsed light for the selected parameters. The analysis of different techniques allows us to determine the germicidal effect, energy consumption and effective germicidal effect.

For Table 1 at the dose of 5 mJ cm^−2^ the irradiance value from the artificial illumination system is 0.15 mW cm^−2^ (lamp power) over 33 s. The pulsed light equivalence at a frequency of 500 Hz and a duty cycle of 30%, in Mode 1 the irradiance reaches 0.5 mW cm^−2^ more than double to match the value set in the light continues to remain constant time (33 s). In Mode 2, the irradiance is identical (0.15 mW cm^−2^) to the continuous light technique, but the time value changes to 110 s. For a dose of 15 mJ cm^−2^ the irradiance value is 0.15 mW cm^−2^ with a time of 100 s with the continuous light technique. Pulsed light emission at a frequency of 500 Hz and a duty cycle of 50%. In Mode 1, the irradiance is exactly twice as high (0.3) as in continuous light and the time is the same. In contrast, for Mode 2, the irradiance of the illumination system is 0.15 mW cm^−2^ and the emission time is twice as long (200 s). Finally, at a dose of 25 mJ cm^−2^ with an irradiance of 0.150 a for a time of 167 s. Mode 1 at a frequency of 500 Hz with a duty cycle of 80% has an irradiance of 0.187 mW cm^−2^ during 167 s. On the other hand, for Mode 2 with the same parameter settings, an irradiance value of 0.150 mW cm^−2^ for 208.7 s is obtained. This means that it requires less time since the pulse is wider. In general, the values obtained will depend on the characteristics of the artificial lighting system and the initial parameter settings such as time, frequency, and duty cycle for pulsed light emission.

### 3.2. Germicidal Effect (GE)

For the programmed doses (5, 15, and 25 mJ cm^−2^), the germicidal effect produced by the continuous and pulsed light techniques was calculated. Table 2 presents the data organized concerning the dose applied on fresh tomatoes in continuous and pulsed light techniques (Mode 1 and Mode 2) for different duty cycle (30%, 50%, and 80%), and frequency at 500 Hz. 

Aerobic Mesophile bacteria count enabled the evaluation of the germicidal effect (GE) for each applied technique. AM integrates the population of bacterial colonies that correspond to cocci, bacilli, and spiral bacteria, that determine the hygienic conditions in food [29,30].

When assessing the effectiveness of the techniques applied, at 5 mJ cm^−2^ the best was the CL compared to the pulsed light techniques in all duty cycles at 30% duty cycle. Mode 2 gave the worst results obtaining a lower performance with the emission of pulsed light in its different duty cycles with a small dose (5 mJ cm^−2^) applied. With the duty cycle configured at 30%, at dose of 15 mJ the CL technique stands out with the highest germicidal effect, with slightly lesser pulsed light in Mode 1 (0.74 LRV) and the last Mode 2. At 25 mJ cm^−2^, the best technique was Mode 1 (1.06) and the least efficient Mode 2. At 50% duty cycle the best treatment was at 25 mJ cm^−2^ dose in pulsed light Mode 1 (1.06 LRV) and the lowest was at 5 mJ cm^−2^ in Mode 2. With an 80%, the best technique was Mode 1 (1.08 LRV) at dose 25 mJ cm^−2^ and the less efficient the Mode 2 (0.90 LRV). 

The statistical analysis of the different light techniques proposed indicates that there exists a significant difference in the three established doses and the light techniques. The next step evaluated the differences in the CL technique and the two pulsed light techniques (Mode 1 and Mode 2). It was found that there are only significant differences between CL and Mode 2. After that, we evaluated the differences in the duty cycle configurations in Mode 1 and Mode 2, and it was found that there are none. Finally, the statistical analysis allowed us to evaluate if there is a lower or higher germicidal effect between a duty cycle with the three different techniques proposed, finding that there is difference. 

Concerning energy consumption, in the 30% duty cycle at a dose of 25 mJ cm^−2^, Mode 1 saved 12%, being the best in terms of germicidal effect. At 50% duty cycle, the consumption in the Mode 1 pulsed light technique saves 11% at a dose of 25 mJ cm^−2^. Finally, the lowest energy consumption of 6% was presented at an 80% duty cycle at a dose of 25 mJ cm^−2^. That is, if someone wants to apply Mode 1 obtains GE and energy consumption. 

Fresh tomato with the third dose (25 mJ cm^−2^) showed enhance germicidal efficacy in the microorganism’s wads. 

In general, the technique applied had a short duration, and there were no changes in the temperature of the product. The germicidal effect evaluated in each of the techniques can be lower due to the amount of irradiation applied to the tomatoes. That is, the amount of artificial UV-C LED irradiation depends on the results obtained in the germicidal effect. 

Although, the irradiance per second and the exposure time are the same, the pulses must have a higher peak power to compensate for the times when the lamp is off. In future works it will be possible to determine the minimum irradiation to achieve an optimal germicidal effect.

### 3.3. Effective Germicidal Effect (EGE)

**Figure 3:**
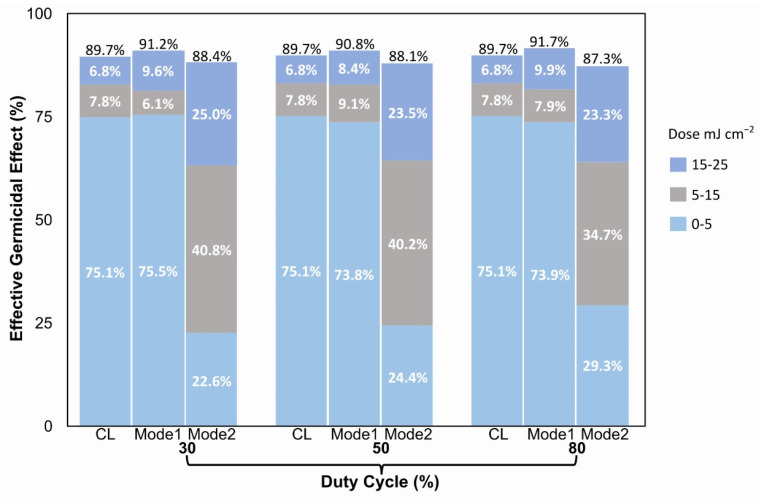
Effective germicidal effect according the dose and light technique applied.

Figure 3 presents the continuous and pulsed UV-C LED performance for the doses applied to fresh tomatoes through the EGE concept. The obtained information displayed in columns according to the dose range and the amount of the microorganisms’ destruction percentage. 

The CL technique generated a 75.1% corresponds to the 0–5 mJ cm^−2^ dose, 7.8% at 5–15 mJ cm^−2^, and 6.8% at the 15–25 mJ cm^−2^ dose. At 30% duty cycle, Mode 1 showed an EGE of 75.5% slightly higher than CL in a range of 0–5 mJ cm^−2^. At dose range of 5–15 mJ cm^−2^, a 6.1%, and 9.6% for a dose of 15–25 mJ cm^−2^, that is, the most significant disinfection occurs in the first dose interval. It may not be necessary to continue applying the rest doses range. Mode 2, 22.6% at the first dose 0–5 mJ cm^−2^, 40.8% for 5–15 mJ cm^−2^, 25.0% in 15–25 mJ cm^−2^. The Mode 2 showed the worst performance during the 30% duty cycle. 

During the 50% duty cycle for the first range of 0–5 mJ cm^−2^ a 75.1% is obtained, 7.8% at 5–15 mJ cm^−2^, and 6.8% at 15–25 mJ cm^−2^ with CL. Mode 1 presented a 73.8%, 9.1%, and 8.4% for 0–5, 5–15, and 15–25 mJ cm^−2^, correspondingly. For Mode 2, with 24.4, 40.2, and 23. 5% for the ranges 0–5, 5–15, and 15–25 mJ cm^−2^, respectively, it shows that the highest disinfection occurs in the first dose range (0–5) with the CL and Mode 1 technique.

At 80% duty cycle, 75.1% at 0–5 mJ cm^−2^ range, 7.8% at range 5 and 15 mJ cm^−2^, and finally 6.8% for 15–25 mJ cm^−2^ for CL technique. Mode 1 presented a 73.9%, 7.9%, and 9.9% in 0–5, 5–15, and 15–25 mJ cm^−2^, correspondingly. Mode 2 with an EGE, at each range 29.3, 34.7 and 23.3% for 0–5, 5–15 and 15–25 mJ cm^−2^, respectively.

With all duty cycles occurred the same behavior, the highest amount of microorganism reduction occurs in the first dose range by CL and Mode 1 techniques standing out.

This concept allows us to identify whether the application of higher doses is required to obtain a higher percentage of destruction of microorganisms. In general, the EGE parameter in the experiment demonstrates in which dose range the highest inactivation of microorganisms occurs with less energy consumption. Additionally, the EGE concept lets us determine whether to continue with the fresh produce disinfection or to stop if the highest germicidal effect has already been achieved in a certain period. That is, although we do not achieve 100% disinfection, we save energy with the artificial radiation system, as well as other types of resources, and that is the purpose. The EGE determine how much energy is consumed to eliminate each percentage, being the germicidal effect during the first dose applied. In our proposal, in the first 5 mJ cm^−2^ dose, 75% of bacteria were eliminated. In the last 10 mJ cm^−2^, only 6% of bacteria were eliminated.

Additionally, it indicates the performance of each technique configured in the artificial illumination system. EGE during dose application represents an important parameter when establishing new methods or procedures for food disinfection. EGE is an optimization factor in any strategy configured in artificial lighting systems and the resources applied, such as energy consumption.

The applied UV-C LEDs was superior in comparison with mercury vapor lamps [23]. According to the results obtained in the research, the PL (Mode1) presents good results in the germicidal effect, in comparison with the CL technique; however, the effectiveness of UV-C LEDs will depend on the type of microorganism, the surface, and even the amount of energy absorbed [11,13,15]. Similarly, the performance is almost the same with some microorganisms, but by optimizing the energy consumption of the lamps. Other studies indicate that the performance of the PL technique is affected by the shadow effect and by the lack of optimization in the frequency and duty cycles [12]. However, the application of pulsed light contributes to the reduction of heating in the lamps, generating even higher performance [23]. In addition, the application of UV-C LEDs would be beneficial for the agro-industrial sector in different processes of the supply chain. Additionally, although there are new proposals further to continuous light use [26,27], the reports where UV-LED with pulsed light has been applied have been mostly for water disinfection. Another point in favor of the techniques presented can be implemented to various processes within the supply chain, such as production, storage, and distribution, to mention a few. The concept of EGE allows us to optimize different resources, such as electricity consumption and the amount of energy that will give us the guideline to decide whether or not to continue with the application of radiation for the inactivation of microorganisms.

## 4. Conclusions

The UV-C LEDs is a tool for food contamination as an alternative to traditional techniques (mercury and xenon lamps) for food disinfection. Furthermore, being compact compared to traditional lamps, they can be integrated into any artificial lighting system with an innovative design. The UV-C LEDs on/off us capability operates immediately, so it does not require a warm-up time which is a common limitation of mercury vapor lamps. Additionally, they are configured to choose a specific wavelength, the most suitable for maximum light absorption for inactivation of microorganisms. 

This proposal provides a mathematical model that serves as a reference to establish the equivalence in the doses applied. New evidence is introduced on the germicidal effect of UV-C LEDs as an alternative method for food disinfection. In addition, it shows that the UV-C pulsed light technique can be a great tool for other areas. 

According to the experiments performed in this proposal, Mode 1 presented the best performance in the germicidal effect, however, between Mode 1 and Mode 2, there is a big difference in the disinfection of fresh produce. The energy consumption measurement in each treatment for the different techniques used is an advantage that allows us to foresee the optimization of resources, showing the best technique for energy saving at the same germicidal effect as CL. The niche remains to continue investigating the different pulsed light techniques towards other applications different than water, or the synergy of different wavelengths to achieve better inactivation of microorganisms. In addition, UV-C LEDs in pulsed light emission can help to avoid the lamps heating, which is always related to the CL, additionally improving thermal management and increasing the germicidal effect on fresh and/or processed products.

## Figures and Tables

**Table 1:** Obtained values for irradiance and time for the proposed techniques.

	Continuous Light			Pulsed Light			
Dose (mJ cm^−2^)	Irradiance (mW cm^−2^)	Time (s)	Duty Cycle(%)	PPL (Mode 1)		TPL (Mode 2)	
				Irradiance (mW cm^−2^)	Time (s)	Irradiance (mW cm^−2^)	Time (s)
5	0.15	33	30	0.50	33	0.15	110
15	0.15	100		0.50	100	0.15	333
25	0.15	167		0.50	167	0.15	556.1
5	0.15	33	50	0.30	33	0.15	66
15	0.15	100		0.30	100	0.15	200
25	0.15	167		0.30	167	0.15	334
5	0.15	33	80	0.187	33	0.15	41.3
15	0.15	100		0.187	100	0.15	125
25	0.15	167		0.187	167	0.15	208.7

**Table 2:** Log reduction values (LRV) and energy consumption (EC) of UV-C LEDs light techniques. Different lowercase letters in the same row indicate statistically significant differences (a, b: *p* < 0.05). Different capital letters in the same column indicate statistically significant differences (A, B, C: *p* < 0.05).

	Continuous Light			Pulsed Light			
Dose (mJ cm^−2^)	CL	EC (Ws)	Duty Cycle (%)	Mode 1	EC (Ws)	Mode 2	EC (Ws)
5	A/a 0.62 ± 0.06	0.22	30	A/aa 0.61 ± 0.03	0.19	A/bb 0.15 ±0.01	0.46
15	B/a 0.77 ± 0.03	0.65		B/aa 0.74 ± 0.01	0.58	B/bb 0.44 ± 0.01	1.39
25	C/a 0.99 ± 0.02	1.09		C/aa 1.06 ± 0.01	0.97	C/bb 0.94 ± 0.01	2.32
5	A/a 0.62 ± 0.06	0.22	50	A/aa 0.60 ± 0.02	0.19	A/bb 0.12 ± 0.02	0.33
15	B/a 0.77 ± 0.03	0.65		B/aa 0.77 ± 0.01	0.59	B/bb 0.45 ± 0.02	1.00
25	C/a 0.99 ± 0.02	1.09		C/aa 1.06 ± 0.01	0.98	C/bb 0.92 ± 0.02	1.66
5	A/a 0.62 ± 0.06	0.22	80	A/aa 0.58 ± 0.01	0.20	A/bb 0.11 ± 0.01	0.25
15	B/a 0.77 ± 0.03	0.65		B/aa 0.74 ± 0.01	0.62	B/bb 0.44 ± 0.01	0.74
25	C/a 0.99 ± 0.02	1.09		C/aa 1.08 ± 0.01	1.03	C/bb 0.90 ± 0.02	1.22

## Data Availability

The data are available from the corresponding author.
